# Absolute and Relative Reliability of the Assessment of the Muscle Mechanical Properties of Pelvic Floor Muscles in Women with and without Urinary Incontinence

**DOI:** 10.3390/diagnostics11122315

**Published:** 2021-12-09

**Authors:** Daiana Priscila Rodrigues-de-Souza, Sandra Alcaraz-Clariana, Lourdes García-Luque, Cristina Carmona-Pérez, Juan Luis Garrido-Castro, Inés Cruz-Medel, Paula R. Camargo, Francisco Alburquerque-Sendín

**Affiliations:** 1 Department of Nursing, Pharmacology and Physical Therapy, Faculty of Medicine and Nursing, University of Córdoba, 14004 Córdoba, Spain; drodrigues@uco.es (D.P.R.-d.-S.); m72alcls@uco.es (S.A.-C.); lgarcial05@hotmail.com (L.G.-L.); mcarperes@yahoo.es (C.C.-P.); inscrz@gmail.com (I.C.-M.); 2 Department of Computer Science and Numerical Analysis, Rabanales Campus, University of Córdoba, 14071 Córdoba, Spain; cc0juanl@uco.es; 3 Maimonides Biomedical Research Institute of Cordoba (IMIBIC), 14004 Córdoba, Spain; 4 Laboratory of Analysis and Intervention of the Shoulder Complex, Department of Physical Therapy, Universidade Federal de São Carlos, Rodovia Washington Luis km 235, São Carlos 13565-905, SP, Brazil; prcamargo@ufscar.br

**Keywords:** muscle tone, tissue stiffness, pelvic floor disorders, young women

## Abstract

An analysis of the muscle mechanical properties (MMPs) of the pelvic floor muscles (PFMs) is relevant for understanding the physiopathology of urinary incontinence (UI). However, there is no objective and reliable methodology currently available for quantifying the MMPs of PFMs. Thus, the objective was to determine the intra-rater and inter-rater reliability of the MMPs of PFM assessment with a hand-held tonometer device, called the MyotonPRO, in young women with and without UI. Sociodemographic and pelvic floor questionnaires, plus MMPs of PFMs were assessed in 38 nulliparous women with UI and 40 matched healthy women by two trained physiotherapists on two different occasions, 48–72 h apart. Good to excellent absolute reliability was found for tone, stiffness, and decrement of both intra- and inter-rater analyses in both study groups (Intraclass Correlation Coefficient ranged from 0.75 to 0.92), with a trend of lower values for relaxation and creep. The standard error of measurement (SEM) did not achieve 10% of the mean values for any MMPs. The minimum detectable change (MDC) values were also provided for clinical applications. In conclusion, the relative reliability of tone, stiffness, and the assessment of the decrement of PFMs with MyotonPRO is good to excellent for UI and healthy women. The SEM and MDC values were acceptable for their application in clinical settings.

## 1. Introduction

The pelvic floor (PF) is a complex set of connective tissues and striated muscles that simultaneously counteract inertial forces and intra-abdominal pressures while maintaining the position of the pelvic organs [1]. The pelvic floor muscles (PFMs) support the pelvic organs through coordinated contraction and relaxation. An increase in intra-abdominal pressure reflexively contracts the PFM, closing the vaginal, urethral and anal hiatus [2]. One of the consequences of the deterioration of these mechanisms is urinary incontinence (UI) [3], which negatively impacts on quality of life and limits activities of daily living [4]. There are several well-known risk factors for UI, such as advanced age, delivery, chronic cough, constipation, high BMI, impact exercise, or having suffered from infantile enuresis [5]. Despite this, incontinence has been detected in young women, with a prevalence of between 8% [6] and 14.3% [7], and 22.9% in physically active women [4]. The most common type of incontinence in this population is stress urinary incontinence (SUI), defined as the involuntary loss of urine that occurs through physical exertion, such as sneezing or coughing [8], and which exceeds 60% of the total UI cases reported in this female population, although mixed UI and urgency UI are also found [4]. In fact, the effect of risk factors for SUI on the PFMs may not be evident until later stages in life [9,10,11], which increases the relevance of the evaluations of PFM, even in subclinical phases.

The relevance of the pelvic girdle biomechanics and the related tissues for SUI has been supported [12]. In fact, there are strong associations between muscle damage and subsequent PF disorders. However, less is known about material physical properties, effects of pregnancy and/or ethnicity on PFMs stiffness, even during pregnancy and at postpartum stage [13]. Commonly, the PFM analysis has focused on strength, evaluated with dynamometry [14], and muscle activity, assessed with electromyography [15]. However, these methodologies do not provide an adequate measurement of muscle tone and other muscle mechanical properties (MMPs), since they do not provide a direct measure of resistance to change in muscle length [16]; in addition, it is invasive and shows limited clinical applicability. In fact, the assessment of the tone of PFM is still based on subjective findings by expert clinicians [17,18], and it has been suggested that it is time to rethink these methods using digital palpation for the assessment of muscle stiffness [19].

Moreover, it is important to recognize that, in order to improve muscle strength, the first choice treatment in UI is rehabilitation of the PF [20,21]. However, there is a lack of reliable instruments for quantifying PFM tone and stiffness [22]. Furthermore, a systematic review in 2015 stated the need for specific methods to assess and distinguish between active (contractile) and passive (viscoelastic properties) components of the MMPs in PFMs [23]. The lack of a reliable and objective method for assessing MMPs of PFMs limits the understanding of the relationship between muscle tone and PF disorders and treatments [16].

Recently, the MyotonPRO, a hand-held, non-invasive device designed to measure MMPs, has shown good clinical applicability and sufficient validity [24] and reliability [25,26] in different contractile [27] and non-contractile [28] tissues and disorders. Regarding PFM, only one study has determined the reliability of the MyotonPRO assessments. This study showed high reliability for adult women with and without vulvodynia; however, no information is available in other clinical stages and populations [16].

Therefore, the aim of this study was to determine the intra-rater and inter-rater reliability for assessing MMPs of PFM with the MyotonPRO device in young women with and without UI.

## 2. Materials and Methods

### 2.1. Study Design

A clinical measurement study assessing absolute and relative reliability using a two-stage repeated measures design was conducted between March 2019 and August 2021. The Guidelines for Reporting Reliability and Agreement Studies (GRAAS) were used [29].

### 2.2. Participants

Participants were women, recruited using a non-probabilistic sampling of consecutive cases via flyers posted on the university campus and social media. Women aged between 18 and 40 years old, and nulliparous, were included if they had any type of UI diagnosed by a physician and confirmed by the 3 Incontinence Questions (3IQ) questionnaire [30,31].

The exclusion criteria were: women currently receiving treatment or seeking treatment for PF or continence; any type of systemic disease that interferes with PF physiology and urination (e.g., multiple sclerosis, lumbopelvic surgery); use of medications that could interfere with continence or muscle performance; BMI greater than 40 kg/m^2^.

The healthy group included women without UI or any lumbopelvic disorder, who did not present any exclusion criteria, and who were matched to urinary incontinent women by age (±3 years) and BMI (±3 kg/m^2^). For two UI women, two matched controls were selected, because of difficulties attending the second assessment session.

A physical therapist with more than five years of experience assessed the inclusion and exclusion criteria. The Research Ethics Committee of Córdoba approved this project (registration number 4074, 20 December 2018 approved). All participants signed the informed consent form.

### 2.3. Assessments and Procedures

The evaluation of each subject was completed in approximately 30 min. Sociodemographic and clinical data were collected, and validated questionnaires commonly used in clinical settings for PF assessment were applied. The Spanish version of the Pelvic Floor Distress Inventory (PFDI-20) includes 20 questions divided into three scales according to the symptoms: symptoms of genital prolapse, questions 1 to 6 (POPDI-6); colorectal-anal symptoms, questions 7 to 14 (CRADI-8); and urinary symptoms, questions 15 to 20 (UDI-6). Each question can be answered on four levels of dysfunction: not at all, somewhat, moderately, or quite a bit. The minimum score for each block is 0 points (minimum dysfunction) and the maximum 100 points (maximum dysfunction). The total score is the sum of the three blocks with a maximum score of 300 [32,33]. The Spanish version of the Pelvic Floor Impact Questionnaire (PFIQ-7) includes seven questions about the impact of symptoms on activities, relationships, or feelings in relation to urinary prolapse (UIQ), colorectal-anal conditions (CRAIQ) and genital conditions (POPIQ). Again, each of the questions can be answered at four levels of participation: not at all, somewhat, moderately, or quite a bit. The minimum score for each block is 0 points (low implication) and the maximum is 100 points (maximum effect). The total score is the sum of the three blocks with a maximum score of 300 [32,33].

**Figure 1:**
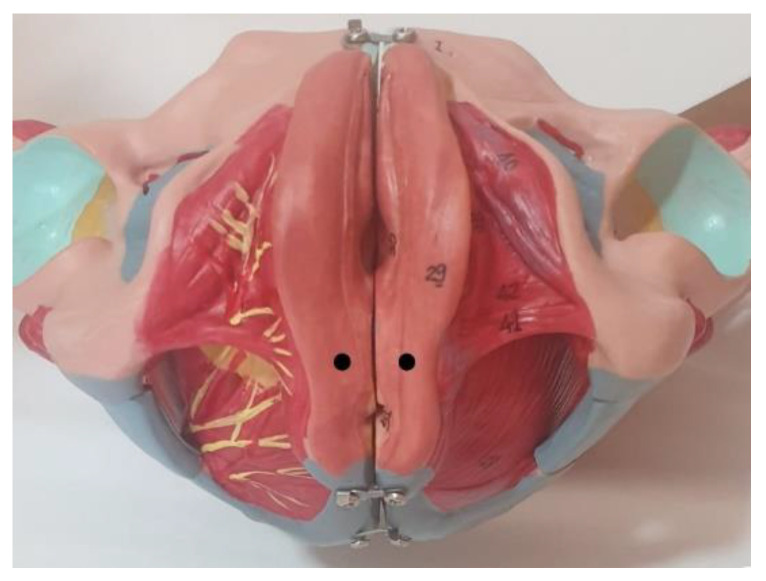
Anatomic location for the MMPs assessment (black dots).

**Figure 2:**
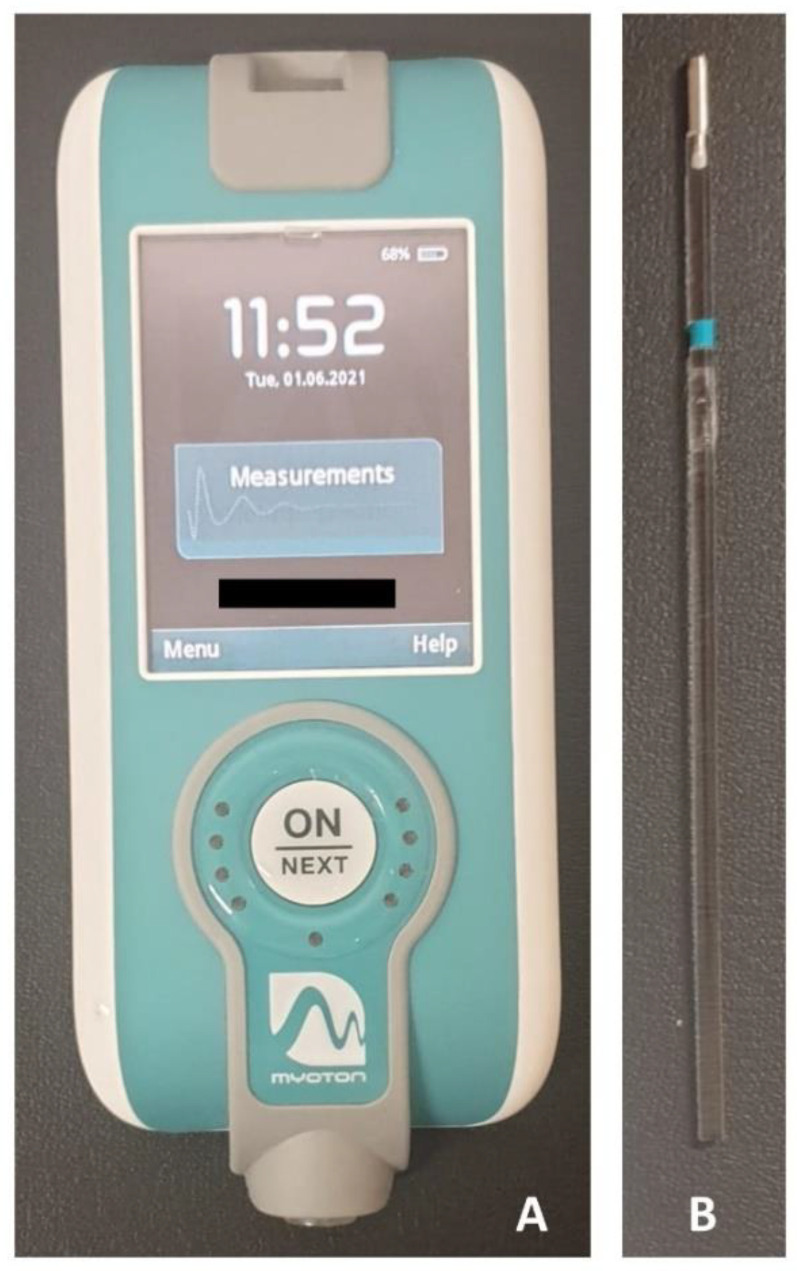
MyotonPro^®^ device (**A**) and 100 mm long probe (**B**).

Subsequently, the evaluation of the MMPs of the PF was carried out. Measurements for the perineal muscles were taken with the participants in a supine position on a table, with the knees flexed and the soles of the feet on the table, to ensure that both lower limbs were symmetrical and relaxed during the measurement. Immediately after a maximal voluntary contraction, and once the muscle was in a relaxed state, the measurement site was located by visual observation and palpation in the largest area of muscle bulk during contraction on both sides of the central perineal body (Figure 1). This area was selected because it contains the most contractile portion of the perineal muscles. A dermographic marker was placed to ensure that the assessors took the measurement at the same site [16]. A manual tonometer (MyotonPro^®^ Myoton AS, Tallinn, Estonia) was used to record the MMPs on the PF (Figure 2A). A 100 mm long probe was used and placed perpendicular to the surface of the skin, directly on the measurement location, and was held while the device performed the predefined series of measurements. According to the manufactures instructions, the test was repeated if the coefficient of variation was higher than 3% among the mechanical impulses [34,35].

The probe exerts mechanical impulses, with a pulse of 15 ms and 0.40 N of mechanical force, to record the tissue response. The average value of three mechanical pulses was used for the analysis. To reduce the abdominal influence on the test, the recording was performed during five seconds of apnea after exhalation [36]. A randomization online tool (www.randomization.com, accessed on 20 February 2019) was used to establish the order of the evaluations (right/left). At the time of each assessment, the assessor was blinded to the previous measurement values.

The MMPs were collected on two different occasions, 48–72 h apart. Inter-rater reliability was determined on the first day, in a randomized order (www.randomization.com, accessed on 20 February 2019) of the raters, and with both assessments performed less than 5 min apart [16]. On the second day, the MMPs of the participants was re-evaluated to determine intra-rater reliability. The rater who repeated the evaluation was a physiotherapist (D.P.R.-d.-S.) with over 10 years of experience in the evaluation and treatment of PF dysfunctions, whereas the second evaluator was a physiotherapist (I.C.-M.) with one year of clinical experience. Both raters were blinded to the clinical condition of the participants, and received six hours of training for the use of the MyotonPRO, to assess muscles of different body regions researched by our team [27,37], including the PFMs.

The description of the MMPs collected by the MyotonPRO is reported elsewhere [38], and can be summarized as: frequency, characterizing muscle tension or tone in resting state (Hz); biomechanical properties, such as stiffness (N/m) and logarithmic decrement in the amplitude of oscillation (Ø), characterizing the inverse of the elasticity [39,40]; and viscoelastic properties, such as relaxation time of stress (ms), and creep (Deborah Number -De-) [41].

A Visual Analogue Scale (VAS) was used to identify the presence and intensity of pain during the evaluation. The presence of pain was monitored to interrupt the patient’s participation in the project if necessary.

### 2.4. Statistical Analysis

The software Tamaño de la Muestra 1.1 (Pontificia Universidad Javeriana, Bogotá, Colombia) was used for sample size calculations. Considering a significance level of 0.05, an Intraclass Correlation Coefficient (ICC) of 0.80 and a confidence interval range of 20%, a sample of 27 individuals was deemed necessary. To account for a withdrawal rate of 25%, at least 40 individuals were included in the study for each group.

Quantitative data were described by the mean and standard deviation on 95% confidence interval (95% CI). All variables showed a normal distribution of data (Kolmogorov-Smirnov test *p* > 0.05). Age, BMI, and questionnaire scores were compared with unrepeated Student *t*-tests. Frequencies and percentages were used to describe qualitative data.

The relative reliability for the measurements of MMPs was determined by calculating ICCs for intra-rater (ICC_2,1_) and inter-rater (ICC_2,2_) reliability. Intra-rater reliability was estimated using the data of the two separated sessions obtained by the same rater, and inter-rater reliability was calculated based on the assessment performed on the first session. For all analyses, the ICC values were considered as poor under 0.5, moderate between 0.51 and 0.75, good between 0.76 and 0.90, and excellent above 0.90 [42]. Furthermore, the means of the MMPs for each reliability analysis were compared using repeated Student *t*-tests.

The absolute reliability was determined according to the Weir approach [43], by calculating the standard error of measurement (SEM) (SEM = SDpooled√1-ICC), and the minimal detectable change (MDC) (MDC_90_ = SEM × √2 × 1.64). The SEM reflects the amount of measurement error for assessments both in the intra- and the inter-rater analyses. The percentage of each SEM in relation to the mean value of each MMP result was also calculated (SEM%). Finally, the MDC is an estimate of the smallest amount of change between assessments that can be detected as true change outside of the measurement error. The MDC helps clinical decision making, such as whether the individual performance represents real change [43,44].

All contrasts were bilateral and *p* < 0.05 was considered significant. Statistical analysis was performed using the IBM SPSS Statistics version 25 (SPSS Inc., Chicago, IL, USA).

## 3. Results

Sociodemographic and clinical data of both groups are included in Table 1. SUI was reported by 60.5% of the sample. There were no differences between groups in age or BMI. On the contrary, PFDI-20 and PFIQ-7 showed differences in the scores when both groups were compared. No individual reported pain (VAS = 0) or any discomfort at the end of the assessment on any of the data collection days.

For intra-rater analysis, relative reliability measures reflected stable MMPs values in both groups and sides, with good to excellent ICC values, which ranged from 0.75 to 0.94, except for the moderate values found for relaxation on the right side for the healthy group, and for creep on the left side for both groups and on the right side for the healthy group (ICC from 0.63 to 0.70). Furthermore, the frequency, stiffness, and relaxation on the left side of the UI group for intra-rater reliability showed statistical differences.

The SEM% ranged from 2.46% to 9.32% for all MMPs, and were lower for the UI group than for the healthy group, with the exception of frequency on the left side. The MDC_90_ was below 2 Hz, while the stiffness achieved 52.34 N/m in the healthy group. Furthermore, for decrement and relaxation, the MDC_90_ for the UI group was ≈50% than for the healthy group on both sides, with the creep between 0.10 and 0.16 for both groups and sides (Table 2).

For inter-rater analysis, relative reliability measures again reflected stable MMP values in both groups and sides, with good to excellent ICC values, ranging from 0.75 to 0.92, with the exception of the moderate value found for relaxation on the right side of the healthy group (ICC = 0.70), and the poor values for creep on both sides and groups (ICC from 0.40 to 0.46). In this case, frequency, relaxation, creep on the left side of the UI group, and relaxation and creep on the left side of the healthy group for inter-rater reliability, showed statistical differences.

The SEM% ranged from 3.12% to 9.06% for all MMPs, without a defined trend of higher values in any group. The MDC_90_ achieved 2.07 Hz in the UI group, while stiffness was below 50 N/m in both groups and sides. Decrement and creep showed stable MDC_90_ values, from 0.12 to 0.16 and from 0.15 to 0.20, respectively. Finally, the relaxation MDC_90_ was over 1.95 ms for all assessments (Table 3).

## 4. Discussion

The present study showed good to excellent intra-rater and inter-rater reliability for the determination of MMPs, and specifically for measurement of tone and biomechanical properties, such as stiffness and decrement, in PFMs. Although several MMPs, mainly in the healthy group, showed statistical differences in both intra- and inter-rater comparisons, all ICC values demonstrated good to very good relative reliability, with the exception of the ICC of relaxation and creep, mainly for inter-rater reliability, which were poor to moderate and also showed statistical differences. Furthermore, SEM and MDC values were also provided for their applications in clinical setting. The percentage of SEM did not achieve 10% of the mean for any MMP or group. Regarding the representativity of the sample, the proportion of UI types was similar than those previously reported in young women [4]. The values of PF distress and impact of the current sample were lower when compared to previous research in women with PF disorders and healthy women [45,46], probably because the current sample was not in treatment or seeking treatment for PF or continence at the time of the study, and also due to the young age of the sample. Furthermore, no women reported pain or any discomfort throughout the assessments, demonstrating the safety and repeatability of the protocol.

Limited information about the determination of MMPs in PFMs is available. In fact, only one study has applied a similar methodology and aims, in this case for women with vulvodynia and a group of healthy women [16]. The relative reliability values were similar to ours, with moderate to excellent reliability, except for frequency and relaxation on the left side for inter-rater analysis of the vulvodynia group, which were poor, as occurred with the relaxation and creep in our sample. However, although SEM percentages were similar to those found in the current study, higher differences can be identified in the absolute values of the means, the SEM and MDC. Part of these differences can be due to the different clinical state of the samples of both studies; however, this interpretation cannot be applied for the healthy groups, where the women in our sample showed a consistent pattern of higher tone, stiffness and decrement, and lower relaxation and creep for the mean values, the SEM and MDC. Two sources of variability can be proposed for these results. First, we included a maintained apnea during the assessment which could vary the abdominal pressure and, consequently, the tension at perineal level. Second, the mean age of our study sample was eight years younger than the sample studied by Davidson et al. [16], and ageing has been reported to be one of the main determinants of lower tone and stiffness, and higher viscoelastic properties, such as relaxation and creep [27,34].

### 4.1. Relative Reliability of MMPs of PFMs Assessment

The lower values of relative reliability found in viscoelastic properties, mainly in creep, in relation to tone and biomechanical properties, could be due to the passive condition of relaxation and creep. In fact, it has been described that active (stiffness and decrement) and passive (relaxation and creep) components of MMPs have a different behavior in PFMs, even when both components influence muscle tone [23]. Moreover, the determination of the viscoelastic properties is more complex, and depends on the rate and velocity of the muscle stretch [47]. It is possible that the participants modified their position between the evaluations, which could change the stretch position of the PFMs and, subsequently, the viscoelastic properties of the MMPs among the evaluations. Nevertheless, future studies should verify this approach.

In general, slightly lower ICC values were identified for MMPs of PFM compared to other muscles and regions [48,49,50], which may be due to a learning curve that could involve the determination of the MMPs for muscles that are difficult to measure [16]. Some of these difficulties are: the specific localization of the measurement, the stabilization of the MyotonPRO position by the rater during the repetition of the pulses for each measurement, or the variability of the relaxation state of the participants during the entire assessment procedure. Further studies with specific training programs are required to address these concerns, although previous studies have questioned the dependence of training and familiarization sessions on the stability of PFM forces [51].

### 4.2. Absolute Reliability of MMPs of PFMs Assessment

In the present study, the SEM, as an absolute reliability measure, has been interpreted both in absolute values, in the same scale of the measure, and in a percentage of the mean, which facilitates clinical interpretations. The MDC was also reported to provide an approach of the thresholds for meaningful clinical changes [52]. Both relative reliability measures were, in general, higher for the healthy women than for the UI group in the intra-rater evaluation, which can be due to a higher variability of the healthy state.

Compared to other assessment tools for PFM evaluation, our results showed similar SEM percentages than those reported for the assessment of dynamometric passive properties of PFM in postmenopausal women with stress UI [53], healthy nulliparous women [54], and electromyography of the PFM [55]. Furthermore, the magnitude of the absolute reliability results are similar to those previously reported for other musculoskeletal evaluations, such as kinematics [56,57].

### 4.3. Clinical Consideration of Assessing the MMPs of PFMs in Women with and without UI

As previously commented, it is relevant to know the MMPs of the PFMs, even in nulliparous young adult women who do not seek health care for PF disorders, as it is a less studied population [3]. In fact, the ability of PFMs to increase the stiffness of the pelvic ring is relevant in women with pelvic pain [58]. Furthermore, it has been described that higher stiffness of vaginal wall tissues is associated with pelvic organ prolapse [59], associated with lower protein expression of collagen III in the vaginal wall than in asymptomatic controls [60].

Finally, despite the fact that in most current studies the results are positive, several authors have raised doubts about the reliability of MyotonPRO, when used in pathological groups [50] or when used in clinical setting. However, the current results show that tone and biomechanical properties of PFMs can be determined in a reliable fashion, and can be used as an objective reference in the clinical state of these muscles, or even in the identification of pre- or post-intervention effects in UI, as previously reported in vulvodynia [16].

### 4.4. Strengths and Limitations of the Study

To the best of our knowledge, the current study is the first to assess intra-rater and inter-rater reliability for the determination of MMPs of PFMs in women with UI using a hand-held portable tonometer. Some methodological aspects were considered to strengthen its internal validity. First, pain and discomfort levels were monitored throughout the women’s participation in the study, to avoid its influence in reliability. Second, the sample size calculation prevented possible losses of statistical power. Third, both raters were blinded to the clinical condition of the women, and even to the data measured by each other, to avoid possible sources of bias.

Nevertheless, some limitations should be recognized. The phase of the menstrual cycle was not controlled in both groups, although the variations of PFM state may not be important due to the short time between assessments [16]. Furthermore, on the second evaluation day, the mark made by the first rater was missing, therefore the assessment could not be performed in exactly in the same place as on the first day. The analysis by age or other factors, such as the type of UI, was not carried out. Therefore, the results cannot be generalizable for other populations and diseases. Finally, caution is required when interpreting inter-rater evaluations in clinical settings, since worse reliability data were found for inter-rater reliability. Future studies on MMPs of PFMs determinations with reliability purposes should consider different factors and diseases.

## 5. Conclusions

The relative reliability of the assessment of MMPs of the PFMs using a handheld tonometer is good to very good for intra-rater and inter-rater reliability in urinary incontinent and healthy women, which supports its use in women with and without UI. However, caution is recommended for relaxation and creep values, mainly for inter-rater evaluations. In addition, SEM and MDC values are acceptable for their application in clinical settings.

## Figures and Tables

**Table 1:** Sociodemographic and clinical data of the sample.

	UI Group (*n* = 38)	Healthy Group (*n* = 40)	*p*-Value
Age (years)	22.3 ± 2.4	22.6 ± 2.7	0.652
BMI (kg/m^2^)	23.5 ± 3.5	21.4 ± 1.7	0.272
Types of UI	SUI: 23; UUI: 8; MUI: 7		
Time suffering UI (years)	3.2 ± 5.3		
PFDI-20	32.9 ± 30.0	18.6 ± 17.1	0.010
PFIQ-7	18.9 ± 33.0	8.0 ± 10.8	0.048

Values expressed as frequencies or means ± SD. Abbreviations: BMI: body mass index; UI: Urinary incontinence; SUI: stress urinary incontinence; MUI: Mixed urinary incontinence; UUI: urgency urinary incontinence; PFDI: Pelvic Floor Distress Inventory; PFIQ: Pelvic Floor Impact Questionnaire.

**Table 2:** Intra-rater reliability for assessing muscle mechanical properties of the pelvic floor muscles in young women with and without incontinence.

		Session			ICC_2,1_ (95% CI)	SEM (SEM%)	MDC_90_	Session			ICC_2,1_ (95% CI)	SEM (SEM%)	MDC_90_
		UI Group						Healthy Group					
		1st session (*n* = 33)	2nd session (*n* = 33)					1st session (*n* = 36)	2nd session (*n* = 36)				
Right side	Frequency (Hz)	15.03 ± 1.23	15.07 ± 1.02	0.760	0.89 (0.77, 0.95)	0.37 (2.46)	0.86	15.89 ± 1.92	15.45 ± 1.70	0.077	0.81 (0.63, 0.90)	0.79 (5.05)	1.84
	Stiffness (N/m)	222.50 ± 31.90	219.20 ± 29.66	0.461	0.82 (0.62, 0.91)	12.98 (5.88)	30.01	246.39 ± 60.49	237.94 ± 49.13	0.370	0.83 (0.61, 0.93)	22.57 (9.32)	52.34
	Decrement	1.02 ± 0.09	0.99 ± 0.12	0.066	0.78 (0.54, 0.90)	0.05 (4.90)	0.11	1.09 ± 0.19	1.02 ± 0.14	0.015 *	0.75 (0.43, 0.90)	0.08 (8.19)	0.20
	Relaxation (ms)	18.12 ± 1.94	18.46 ± 1.83	0.047	0.94 (0.87, 0.97)	0.46 (2.51)	1.02	16.84 ± 2.26	17.77 ± 2.70	0.051	0.70 (0.40, 0.87)	1.53 (8.84)	3.55
	Creep (De)	1.00 ± 0.10	1.02 ± 0.09	0.230	0.85 (0.68, 0.93)	0.04 (3.77)	0.09	0.93 ± 0.10	0.99 ± 0.14	0.016 *	0.63 (0.36, 0.81)	0.07 (6.95)	0.15
Left side	Frequency (Hz)	15.23 ± 1.54	14.49 ± 1.34	0.010 *	0.86 (0.70, 0.93)	0.55 (3.73)	1.28	16.02 ± 1.57	16.03 ± 1.40	0.954	0.76 (0.42, 0.89)	0.55 (3.44)	1.28
	Stiffness (N/m)	228.00 ± 28.40	215.60 ± 27.81	0.012 *	0.82 (0.61, 0.91)	12.31 (5.55)	28.55	250.28 ± 46.54	250.61 ± 39.25	0.966	0.86 (0.63, 0.95)	22.56 (9.01)	52.33
	Decrement	1.04 ± 0.13	1.00 ± 0.12	0.051	0.77 (0.50, 0.90)	0.06 (5.92)	0.14	1.05 ± 0.19	1.05 ± 0.12	0.848	0.75 (0.41, 0.89)	0.09 (8.79)	0.21
	Relaxation (ms)	17.69 ± 1.87	18.35 ± 1.78	0.004 *	0.88 (0.76, 0.95)	0.64 (3.53)	1.48	16.43 ± 1.70	16.99 ± 1.89	0.045 *	0.85 (0.63, 0.96)	1.20 (7.20)	2.79
	Creep (De)	0.98 ± 0.09	0.98 ± 0.07	0.953	0.68 (0.40, 0.82)	0.05 (4.71)	0.11	0.91 ± 0.07	0.96 ± 0.09	0.002 *	0.66 (0.38, 0.83)	0.07 (7.64)	0.16

Values expressed as means ± SD. * Significant difference (*p* < 0.05) between measurements. Abbreviations: ICC, intraclass correlation coefficient; SEM, standard error of measurement; SEM%, standard error of measurement percentage with respect to the average value; MDC, minimal detectable change. 95% CI, 95% confidence interval.

**Table 3:** Inter-rater reliability for assessing muscle mechanical properties of the pelvic floor muscles in young women with and without incontinence.

		Rater			ICC_2,2_ (95% CI)	SEM (SEM%)	MDC_90_	Rater		*p*-Value	ICC_2,2_ (95% CI)	SEM (SEM%)	MDC_90_
		UI Group						Healthy Group					
		Rater 1 (*n* = 38)	Rater 2 (*n* = 38)					Rater 1 (*n* = 40)	Rater 2 (*n* = 40)				
Right side	Frequency (Hz)	15.02 ± 2.79	15.04 ± 1.29	0.406	0.83 (0.65, 0.95)	0.89 (5.93)	2.07	15.78 ± 1.93	15.39 ± 1.27	0.090	0.89 (0.69, 0.98)	0.53 (3.43)	1.24
	Stiffness (N/m)	224.18 ± 55.96	220.27 ± 38.01	0.265	0.80 (0.63, 0.91)	20.13 (9.06)	46.68	241.74 ± 59.59	232.32 ± 38.97	0.288	0.82 (0.56, 0.95)	19.78 (8.34)	45.88
	Decrement	1.07 ± 0.17	1.04 ± 0.09	0.326	0.78 (0.48, 0.92)	0.07 (6.17)	0.15	1.08 ± 0.19	1.03 ± 0.15	0.054	0.87 (0.65, 0.98)	0.06 (5.87)	0.14
	Relaxation (ms)	17.45 ± 2.84	17.47 ± 1.22	0.953	0.78 (0.47, 0.92)	1.02 (5.84)	2.36	16.99 ± 2.30	17.79 ± 2.03	0.035 *	0.73 (0.40, 0.91)	1.12 (6.46)	2.61
	Creep (De)	0.97 ± 0.14	0.96 ± 0.08	0.682	0.42 (0.15, 0.75)	0.09 (8.85)	0.20	0.93 ± 0.10	0.98 ± 0.10	0.014 *	0.40 (0.11, 0.73)	0.08 (8.14)	0.18
Left side	Frequency (Hz)	15.44 ± 1.62	14.70 ± 1.55	0.040 *	0.85 (0.66, 0.95)	0.63 (4.15)	1.45	15.99 ± 1.53	15.97 ± 1.16	0.892	0.86 (0.64, 0.98)	0.50 (3.12)	1.15
	Stiffness (N/m)	242.18 ± 47.97	222.27 ± 36.26	0.050	0.83 (0.64, 0.93)	17.88 (7.70)	41.48	249.21 ± 55.18	247.79 ± 42.15	0.853	0.81 (0.53, 0.95)	20.98 (8.45)	48.67
	Decrement	1.00 ± 0.17	1.00 ± 0.14	0.124	0.80 (0.50, 0.93)	0.07 (6.95)	0.16	1.04 ± 0.19	1.05 ± 0.17	0.535	0.92 (0.84, 0.96)	0.05 (4.73)	0.12
	Relaxation (ms)	17.51 ± 1.86	18.52 ± 1.71	0.011 *	0.75 (0.49, 0.89)	0.92 (5.12)	2.14	16.49 ± 1.67	17.22 ± 1.82	0.014 *	0.77 (0.46, 0.92)	0.84 (4.98)	1.95
	Creep (De)	0.95 ± 0.09	0.99 ± 0.08	0.026 *	0.44 (0.20, 0.77)	0.07 (6.77)	0.15	0.91 ± 0.07	0.98 ± 0.09	<0.001 *	0.46 (0.20, 0.76)	0.06 (6.63)	0.15

Values expressed as means ± SD. * Significant difference (*p* < 0.05) between measurements. Abbreviations: ICC, intraclass correlation coefficient; SEM, standard error of measurement; SEM%, standard error of measurement percentage with respect to the average value; MDC, minimal detectable change. 95% CI, 95% confidence interval.

## Data Availability

The data presented in this study are available upon reasonable request from the corresponding author.
